# Adaptations in Energy Expenditure Following Caloric Restriction in Older Adults

**DOI:** 10.1093/geroni/igaf122.1019

**Published:** 2025-12-31

**Authors:** Denise Houston, Jason Fanning, James DeLany, Fang-Chi Hsu, Shyh-Huei Chen, Michael Miller, Stephen Kritchevsky, Barbara Nicklas

**Affiliations:** Wake Forest University School of Medicine, Winston-Salem, North Carolina, United States; Wake Forest University, Winston Salem, North Carolina, United States; AdventHealth, Orlando, Florida, United States; Wake Forest University School of Medicine, Winston-Salem, North Carolina, United States; Wake Forest University School of Medicine, Winston-Salem, North Carolina, United States; Wake Forest University School of Medicine, Winston-Salem, North Carolina, United States; Wake Forest University School of Medicine, Winston-Salem, North Carolina, United States; Wake Forest University School of Medicine, Winston-Salem, North Carolina, United States

## Abstract

Adaptations in components of energy expenditure (EE) occur in response to caloric restriction (CR) and contribute to the propensity for weight regain following weight loss. However, the magnitude of changes in EE following CR is not well known in older adults. We examined changes in total EE (TEE by doubly labeled water, DLW) and resting EE (REE by indirect calorimetry) following the 9-month HALLO-P CR interventions. The degree of adaptive thermogenesis (AT; defined as changes in EE beyond what is expected from changes in body size and composition) was also analyzed. Among 56 older adults with overweight/obesity (68±5 yrs; 61% female; 31.6±3.0 kg/m2) who were randomized to CR, the degree of CR over 9 months was estimated as 7.9% based on the intake-balance method with DLW-TEE used to estimate energy intake. This degree of CR decreased body weight by 6.4±5.4 kg (-7.0%) and fat free mass (FFM) by 2.1±1.9 kg (-4.0%). Baseline raw values of TEE and REE were 2327±449 and 1619±297 kcal/day, respectively. Both TEE and REE decreased slightly, but not significantly, following CR (TEE: Δ= -47±353 kcal/d, <0.5%; REE: Δ = -59±218 kcal/d, 3.6%). Adaptive thermogenesis for TEE and REE (adjusted for age, race, sex, and baseline height, weight, fat-free mass, fat mass, and step count) showed non-significant increases after CR (TEE-AT: 48±342 kcal/d; REE-AT: 29±185 kcal/d). We will also report the relationship of these adaptations to changes in body mass and FFM. These results suggest little adaptive thermogenesis to moderate CR over 9 months in older adults.

